# Acute Hyperinsulinemia Alters Bone Turnover in Women and Men With Type 1 Diabetes

**DOI:** 10.1002/jbm4.10389

**Published:** 2020-08-03

**Authors:** Vanessa D Sherk, Timothy Vigers, Laura Pyle, Janet K Snell‐Bergeon, Kristen J Nadeau, Michael R Rickels, Kellee M Miller, Carla J Greenbaum, Viral N Shah

**Affiliations:** ^1^ Department of OrthopedicsSchool of Medicine University of Colorado Anschutz Medical Campus Aurora CO USA; ^2^ Department of Biostatistics and Informatics Colorado School of Public Health University of Colorado Anschutz Medical Campus Aurora CO USA; ^3^ Department of Pediatrics, Section of EndocrinologySchool of Medicine University of Colorado Anschutz Medical Campus Aurora CO USA; ^4^ Barbara Davis Center for Diabetes University of Colorado Anschutz Medical Campus Aurora CO USA; ^5^ Children's Hospital Colorado University of Colorado School of Medicine Aurora CO USA; ^6^ Institute for Diabetes, Obesity & Metabolism University of Pennsylvania Perelman School of Medicine Philadelphia PA USA; ^7^ Jaeb Center for Health Research Tampa FL USA; ^8^ Diabetes Program Benaroya Research Institute Seattle WA USA

**Keywords:** BONE METABOLISM, HYPERINSULINEMIC‐EUGLYCEMIC CLAMP, TYPE 1 DIABETES

## Abstract

Type 1 diabetes (T1D) increases fracture risk across the lifespan. The low bone turnover associated with T1D is thought to be related to glycemic control, but it is unclear whether peripheral hyperinsulinemia due to dependence on exogenous insulin has an independent effect on suppressing bone turnover. The purpose of this study was to test the bone turnover marker (BTM) response to acute hyperinsulinemia. Fifty‐eight adults aged 18 to 65 years with T1D over 2 years were enrolled at seven T1D Exchange Clinic Network sites. Participants had T1D diagnosis between age 6 months to 45 years. Participants were stratified based on their residual endogenous insulin secretion measured as peak C‐peptide response to a mixed meal tolerance test. BTMs (CTX, P1NP, sclerostin [SCL], osteonectin [ON], alkaline phosphatase [ALP], osteocalcin [OCN], osteoprotegerin [OPG], osteopontin [OPN], and IGF‐1) were assessed before and at the end of a 2‐hour hyperinsulinemic‐euglycemic clamp (HEC). Baseline ON (*r* = −0.30, *p* = .022) and OCN (*r* = −0.41, *p* = .002) were negatively correlated with age at T1D diagnosis, but baseline BTMs were not associated with HbA1c. During the HEC, P1NP decreased significantly (−14.5 ± 44.3%; *p* = .020) from baseline. OCN, ON, and IGF‐1 all significantly increased (16.0 ± 13.1%, 29.7 ± 31.7%, 34.1 ± 71.2%, respectively; all *p* < .001) during the clamp. The increase in SCL was not significant (7.3 ± 32.9%, *p* = .098), but the decrease in CTX (−12.4 ± 48.9, *p* = .058) neared significance. ALP and OPG were not changed from baseline (*p* = .23 and *p* = .77, respectively). Baseline ON and SCL were higher in men, but OPG was higher in women (all *p* ≤ .029). SCL was the only BTM that changed differently in women than men. There were no differences in baseline BTMs or change in BTMs between C‐peptide groups. Exogenous hyperinsulinemia acutely alters bone turnover, suggesting a need to determine whether strategies to promote healthy remodeling may protect bone quality in T1D. © 2020 American Society for Bone and Mineral Research © 2020 The Authors. *JBMR Plus* published by Wiley Periodicals LLC on behalf of American Society for Bone and Mineral Research.

## Introduction

Type 1 diabetes (T1D) increases fracture risk across the lifespan, in part due to decreased volumetric bone mineral density (vBMD) and cortical thinning.^(^ ^1^ ^)^ This appears to be particularly true in those with a childhood onset of T1D.^(^ ^2^, ^3^ ^)^ T1D is thought to decrease bone quality via hyperglycemia‐induced reductions in bone turnover.^(^ ^4^, ^5^, ^6^ ^)^ Acute suppression of markers of bone formation and resorption occurs after a meal, oral glucose, and intravenous glucose, indicating that either glucose or endogenous insulin suppresses bone turnover markers (BTMs).^(^ ^7^, ^8^ ^)^ Extending to the context of T1D, chronically low levels of insulin or frequent hyperglycemia may each slow bone turnover or accrual.^(^ ^9^, ^10^, ^11^ ^)^

Exogenous insulin is generally required to maintain glucose control in T1D, which could have both a direct and indirect role in acutely stimulating or suppressing bone turnover. There is evidence in preclinical models that bone and pancreatic β‐cells are linked by a feedback loop by which osteocalcin (OCN) promotes β‐cell production and secretion of insulin.^(^ ^12^ ^)^ Insulin then promotes the secretion of OCN, but this feedforward loop between the pancreas and the skeleton may be impaired with T1D due to a lack of pancreatic β cell function. Because the lowered bone density of T1D in children and adults does not suggest that exogenous insulin supports promotes bone formation, it is important to know what effect insulin has on bone turnover. Studies have shown low IGF‐1 in patients with T1D, which could indirectly affect bone turnover.^(^ ^13^, ^14^, ^15^ ^)^ With impaired endogenous secretion, portal levels of insulin are low in T1D whereas the exogenous insulin results in peripheral hyperinsulinemia. A consequence is that, despite adequate dose of insulin to normalize blood glucose, this does not result in an increase in IGF‐1.^(^ ^16^, ^17^ ^)^ Although controversial, peripheral levels of insulin could increase glucose uptake in bone, thereby stimulating bone turnover.^(^ ^18^, ^19^ ^)^

Osteoblast‐derived BTMs such as OCN and alkaline phosphatase (ALP) have received the greatest amount of focus in understanding reductions in bone quality with diabetes, in part because of the bone–pancreas feedback loop.^(^ ^20^ ^)^ However, because osteocytes are the master regulators of bone remodeling, osteocyte‐derived proteins are more often recognized as potential mediators of reduced bone quality with T1D. Sclerostin (SCL), a glycoprotein secreted by osteocytes, is a well‐known inhibitor of the Wnt/β–catenin pathway and bone formation, and has been reported to be higher in youth with both T1D and T2D.^(^ ^21^, ^22^ ^)^ However, direct link between hyperglycemia or hyperinsulinemia/hypoinsulinemia on SCL secretion is not well‐established. If exogenous insulin acutely increases the secretion of SCL, then it would be a clear target for therapeutic strategies to improve bone health in T1D.

We investigated the acute BTM response to exogenous insulin in adults with T1D during a hyperinsulinemic‐euglycemic clamp (HEC) performed as part of a larger study designed to evaluate the impact of different levels of endogenous insulin secretion measured as C‐peptide on various clinical and metabolic variables. Because exogenous insulin could stimulate the bone–pancreas feedback loop in those with impaired insulin secretion, we hypothesized that the BTMs would have the greatest change in those who were C‐peptide–negative or low, compared to intermediate or high C‐peptide levels. We also compared the BTM response between sexes to determine if exogenous insulin has a sex‐dependent effect on bone turnover in adults with T1D.

## Subjects and Methods

### Subjects

The original study was designed to evaluate β‐cell function and glucose counter regulation during progression of T1D and enrolled 63 subjects aged 18 to 65 years with a T1D duration over 2 years at seven T1D Exchange Clinic Network sites (all sites are listed in the online Supplementary Appendix). Serum samples were unavailable or inadequate for bone marker analysis for five participants, and therefore, 58 adults were included in this study. Participants had a diagnosis of T1D at age 6 months to 46 years. T1D was defined based on clinical diagnosis. An individual must have had a clinical diagnosis of autoimmune type 1 diabetes, as determined by the physician/study investigator, and either islet cell antibodies present or, if antibodies were negative or unknown, then insulin must have been started at or shortly after diagnosis and used continually thereafter (except in the case of a pancreas or islet cell transplant).^(^ ^23^, ^24^ ^)^ Participants had BMI < 30 kg/m^2^, and glycated hemoglobin (HbA1c) <9.0. Exclusion criteria included impaired kidney function, as defined by serum potassium >5.5 mmol/L or serum creatinine >1.4 mg/dL in women or > 1.5 mg/dL in men; impaired liver function, as defined by total bilirubin, aspartate aminotransferase, alanine aminotransferase, or ALP more than two times the upper limit of normal; adrenal insufficiency requiring glucocorticoid replacement; active cardiovascular disease; history of seizure disorder; received treatment with medications that interfere with glucose or islet hormone metabolism other than insulin in the past month; experienced an episode of hypoglycemia or diabetic ketoacidosis in the past 3 months; or anemia defined as hemoglobin <12 g/dL in men or < 11 g/dL in women; or known coagulopathy. Participants came in for the following visits: screening, Mixed Meal Tolerance Test (MMTT) and continuous glucose monitor (CGM) placement, and HEC. The details of the study protocol and procedures was published recently.^(^ ^23^ ^)^ This study was approved by the IRB of seven participating sites.

### MMTT

A 2‐hour MMTT with a standardized liquid meal (Boost High Protein; Nestle HealthCare Nutrition, Inc., Bridgewater, NJ, USA; 6 mL/kg up to 360 mL) was conducted after a 10‐hour overnight fast as an assessment of islet hormone secretion in response to nutrient ingestion.^(^ ^23^ ^)^ The peak C‐peptide in response to the MMTT was used to stratify subjects into four groups based on endogenous insulin secretion^(^ ^15^ ^)^: (i) negative <0.007 pmol/mL (0.02 ng/mL); (ii) low 0.017–0.200 pmol/mL (0.05–0.60 ng/mL); (iii) medium >0.200–0.400 pmol/mL (>0.6–1.20 ng/mL); and (iv) high >0.400 pmol/mL (>1.20 ng/mL) (Table 1).

**Table 1:** Descriptive Characteristics by C‐Peptide Status

	Negative (*n* = 15)	Low (*n* = 14)	Intermediate (*n* = 12)	High (*n* = 17)	*p*
Age (years), mean ± SD	25.9 ± 11.4	28.4 ± 8.2	28.2 ± 9.2	28.7 ± 8.7	.85
Gender (male), *n* (%)	8 (53.3)	9 (64.3)	6 (50.0)	7 (41.2)	.64
Ethnicity, *n* (%)					1.00
Not Hispanic or Latino	14 (93.3)	13 (92.9)	12 (100.0)	15 (88.2)	
Hispanic or Latino	1 (6.7)	1 (7.1)	0 (0.0)	1 (5.9)	
Unknown/not reported	0 (0.0)	0 (0.0)	0 (0.0)	1 (5.9)	
Race, *n* (%)					.98
White	15 (100.0)	13 (92.9)	12 (100.0)	14 (82.4)	
Asian	0 (0.0)	0 (0.0)	0 (0.0)	1 (5.9)	
Black/African American	0 (0.0)	0 (0.0)	0 (0.0)	1 (5.9)	
More than one race	0 (0.0)	1 (7.1)	0 (0.0)	0 (0.0)	
Unknown/not reported	0 (0.0)	0 (0.0)	0 (0.0)	1 (5.9)	
T1D years, median [IQR]	10.0 [8, 13]	6.5 [3, 10]	5.0 [3, 5]	3.0 [2, 5]	<.001
BMI (kg/m^2^), mean ± SD	25.2 ± 3.2	24.4 ± 2.6	23.3 ± 2.9	23.9 ± 3.5	.47
Daily insulin dose (units/kg), mean ± SD	0.72 ± 0.19	0.61 ± 0.18	0.48 ± 0.21	0.49 ± 0.24	.007
HbA1c (%), mean ± SD^a^	7.6 ± 0.8	6.8 ± 0.9	7.0 ± 0.8	6.8 ± 1.0	.052
Insulin pump use, *n* (%)	10 (66.7)	12 (85.7)	6 (50.0)	6 (35.3)	.032
CGM use (yes), *n* (%)	5 (33.3)	6 (42.9)	8 (66.7)	6 (35.3)	.32
Vitamin D (IU), mean ± SD	29.1 ± 6.5	25.2 ± 8.1	24.1 ± 7.6	27.0 ± 7.7	.31
Calcium (mg/dL), mean ± SD	9.4 ± 0.3	9.2 ± 0.4	9.4 ± 0.4	9.4 ± 0.4	.25
Albumin (g/dL), mean ± SD	4.4 ± 0.3	4.4 ± 0.2	4.4 ± 0.2	4.4 ± 0.3	.88
Adjusted Ca (mg/dL), mean ± SD^b^	9.5 ± 0.2	9.3 ± 0.3	9.5 ± 0.4	9.5 ± 0.4	.25
eGFR (mL/min/1.73m^2^), mean ± SD^c^	112.9 ± 26.6	110.8 ± 21.0	103.7 ± 16.9	107.8 ± 25.6	.77
Creatinine (mg/dL), mean ± SD	0.80 ± 0.11	0.83 ± 0.18	0.83 ± 0.13	0.81 ± 0.17	.95

CGM = continuous glucose monitor; eGFR = estimated glomerular filtration rate; HbA1c = glycated hemoglobin; MDRD = Modification of Diet in Renal Disease; T1D = type 1 diabetes.

^a^ To convert to mmol/mol, multiply by 10.93 and subtract 23.50.

^b^ Corrected calcium = (0.8 * [normal albumin – patient's albumin]) + serum Ca. Normal albumin was defaulted to 4 mg/dL.

^c^ eGFR was calculated using the MDRD equation.

### HEC

After a 10‐hour overnight fast, all participants underwent an HEC. Participants were placed on a low‐dose intravenous insulin infusion protocol to target blood glucose 81 to 115 mg/dL prior to testing.^(^ ^25^ ^)^ One catheter was placed in an antecubital vein for infusions, and a second catheter was placed in a distal forearm or hand vein for blood sampling. At t = −120 min, a primed (5 mg/kg fasting plasma glucose of 90 mg/dL was given over 5 min) continuous (0.05 mg/kg/min for 355 min) infusion of 6,6‐^2^H_2_‐glucose (99% enriched; Cambridge Isotopes Laboratories, Andover, MA, USA) was initiated. After baseline blood sampling (−15 min, −1 min, 0 min time points), a primed (1.6 mU/kg/min given over 10 min) continuous (0.8 mU/kg/min) infusion of insulin was administered to produce hyperinsulinemia.^(^ ^26^ ^)^ After initiating the insulin infusion, a variable rate infusion of 20% glucose enriched to 2% with 6,6‐^2^H_2_‐glucose was administered according to the glycemic clamp technique to achieve a plasma glucose of 90 mg/dL by 60 min and maintained until 120 min. Markers of bone turnover were measured at 0 and 120 min of the clamp. The results of the tracer study used to derive measures of hepatic and peripheral insulin sensitivity have been published.^(^ ^23^ ^)^

### BTMs

BTM analyses were performed on the second freeze‐thaw cycle of the blood samples (ie, samples were thawed one time prior to use). Post‐MMTT C‐peptide levels were measured by two‐site immunoenzyomometric assays (Tosoh 2000 auto‐analyzer; Tosoh Bioscience, San Francisco, CA, USA). The C‐peptide assay has a sensitivity level of detection at 0.02 ng/mL. Vitamin D was measured using enzyme immunoassay (EIA) (Immunodiagnostic Systems, Gaithersburg, MD, USA). IGF‐1 was measured using ELISA (Biovendor, Brno, Czech Republic) kit. Serum C‐terminal telopeptides of type I collagen (CTX) was measured using EIA and intact procollagen type 1 N‐terminal propeptide (P1NP) was measured by chemiluminescence (Immunodiagnostic Systems, Boldon Business Park, UK). Osteoprotegerin (OPG), SCL, and ALP were measured using MSD human bone panel‐1 (Meso Scale Diagnostics, Rockville, MD, USA). Osteocalcin (OCN) and osteonectin (ON) were measured using MSD human bone panel‐2. The limits of detection were as follows: IGF‐1 0.09 ng/mL; CTX‐1 0.02 ng/mL; OCN 0.023 ng/mL; ON 2.6 ng/mL; OPN 0.05 ng/mL; OPG 0.004 ng/mL; SCL 0.004 ng/mL; and ALP 1.9 ng/mL.

### Statistical analyses

Data were analyzed using R version 3.5.3 (R Foundation for Statistical Computing, Vienna, Austria; https://www.r-project.org/), and are reported as mean ± SD, unless otherwise denoted. Percent changes in BTMs were calculated and were tested whether they significantly differed from zero. To test the effect of C‐peptide status or sex on the change in BTM during the HEC, the change in BTM was compared between C‐peptide groups with ANOVA, and between sexes with an independent *t* test. Pearson correlation coefficients were used to determine whether baseline BTMs or the change in BTMs were correlated with the age at diagnosis, HbA1c, or changes in insulin. Significance was defined as *p* < .05. All model assumptions were checked and residuals visually assessed.

## Results

The duration of T1D ranged from 2 to 41 years, and 52% were diagnosed by age 18 years. Participants who were C‐peptide negative had a longer duration of T1D, used a higher daily insulin dose, and tended to have the highest HbA1c (Table 1). Women had higher vitamin D (*p* = .020) than men, and men had higher creatinine, estimated glomerular filtration rate (eGFR), and albumin, and used a higher daily insulin dose than women (Table 2). Overall descriptive characteristics of the entire cohort are given in Supplemental Table 1.

**Table 2:** Descriptive Characteristics by Sex

	Female (*n* = 28)	Male (*n* = 30)	*p*
Age (years), mean ± SD	28.1 ± 8.5	27.5 ± 10.1	.83
Ethnicity, *n* (%)			.80
Not Hispanic or Latino	26 (92.9)	28 (93.3)	
Hispanic or Latino	2 (7.1)	1 (3.3)	
Unknown/not reported	0 (0.0)	1 (3.3)	
Race, *n* (%)			1.000
White	27 (96.4)	27 (90.0)	
Asian	0 (0.0)	1 (3.3)	
Black/African American	1 (3.6)	0 (0.0)	
More than one race	0 (0.0)	1 (3.3)	
Unknown/not reported	0 (0.0)	1 (3.3)	
T1D years, median [IQR]	7.6 [5.9]	7.2 [7.1]	.83
BMI (kg/m^2^), mean ± SD	24.2 ± 3.5	24.2 ± 2.8	.99
Daily insulin dose (units/kg), mean ± SD	0.5 ± 0.2	0.6 ± 0.2	.02
HbA1c (%), mean ± SD^a^	7.2 ± 0.9	6.9 ± 0.9	.21
Insulin pump use, *n* (%)	15 (53.6)	19 (63.3)	.63
CGM use (yes), *n* (%)	12 (42.9)	13 (43.3)	1.00
Vitamin D (IU), mean ± SD	28.9 ± 5.6	24.3 ± 8.4	.020
Calcium (mg/dL), mean ± SD	9.3 ± 0.3	9.4 ± 0.4	.42
Albumin (g/dL), mean ± SD	4.3 ± 0.3	4.5 ± 0.2	.015
Adjusted Ca (mg/dL), mean ± SD^b^	9.4 ± 0.3	9.4 ± 0.4	.59
eGFR (mL/min/1.73m^2^), mean ± SD^c^	102.2 ± 21.0	115.0 ± 22.9	.033
Creatinine (mg/dL), mean ± SD	0.76 ± 0.13	0.88 ± 0.14	.001

CGM = continuous glucose monitor; eGFR = estimated glomerular filtration rate; HbA1c = Glycated hemoglobin; MDRD = Modification of Diet in Renal Disease; T1D = type 1 diabetes.

^a^ To convert to mmol/mol, multiply by 10.93 and subtract 23.50.

^b^ Corrected calcium = (0.8 * [normal albumin – patient's albumin]) + serum Ca. Normal albumin was defaulted to 4 mg/dL.

^c^ eGFR was calculated using the MDRD equation.

**Fig 1:**
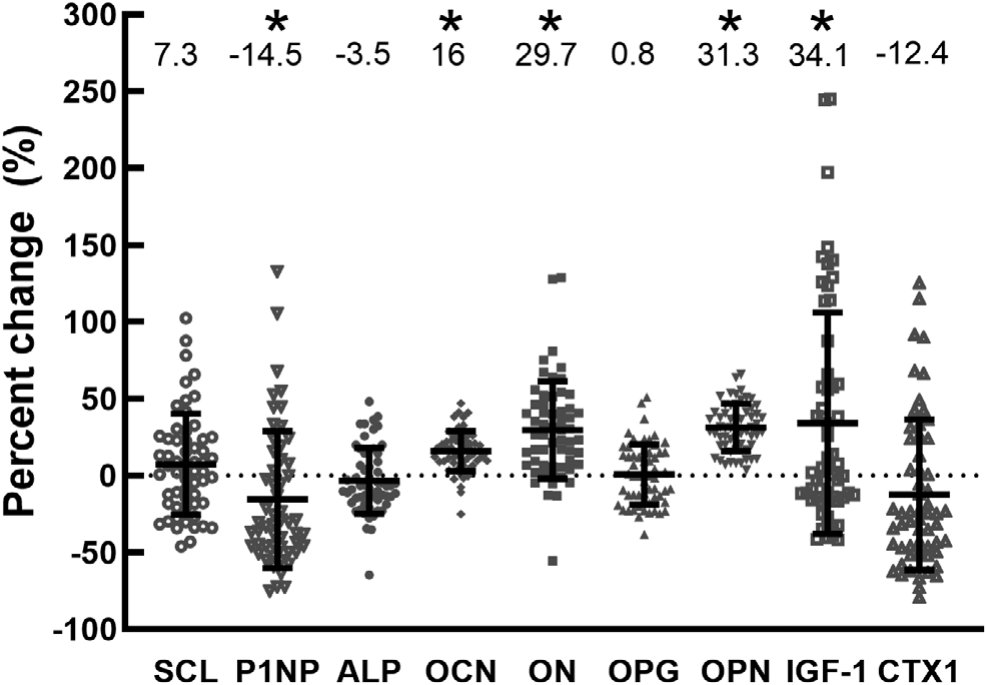
Relative (%) change in bone turnover markers in response to a hyperinsulinemic euglycemic clamp. Values shown in the figure are mean % changes. Individual data points, with mean ± SD. *n* = 58. *p < .05 different from zero.

**Fig 2:**
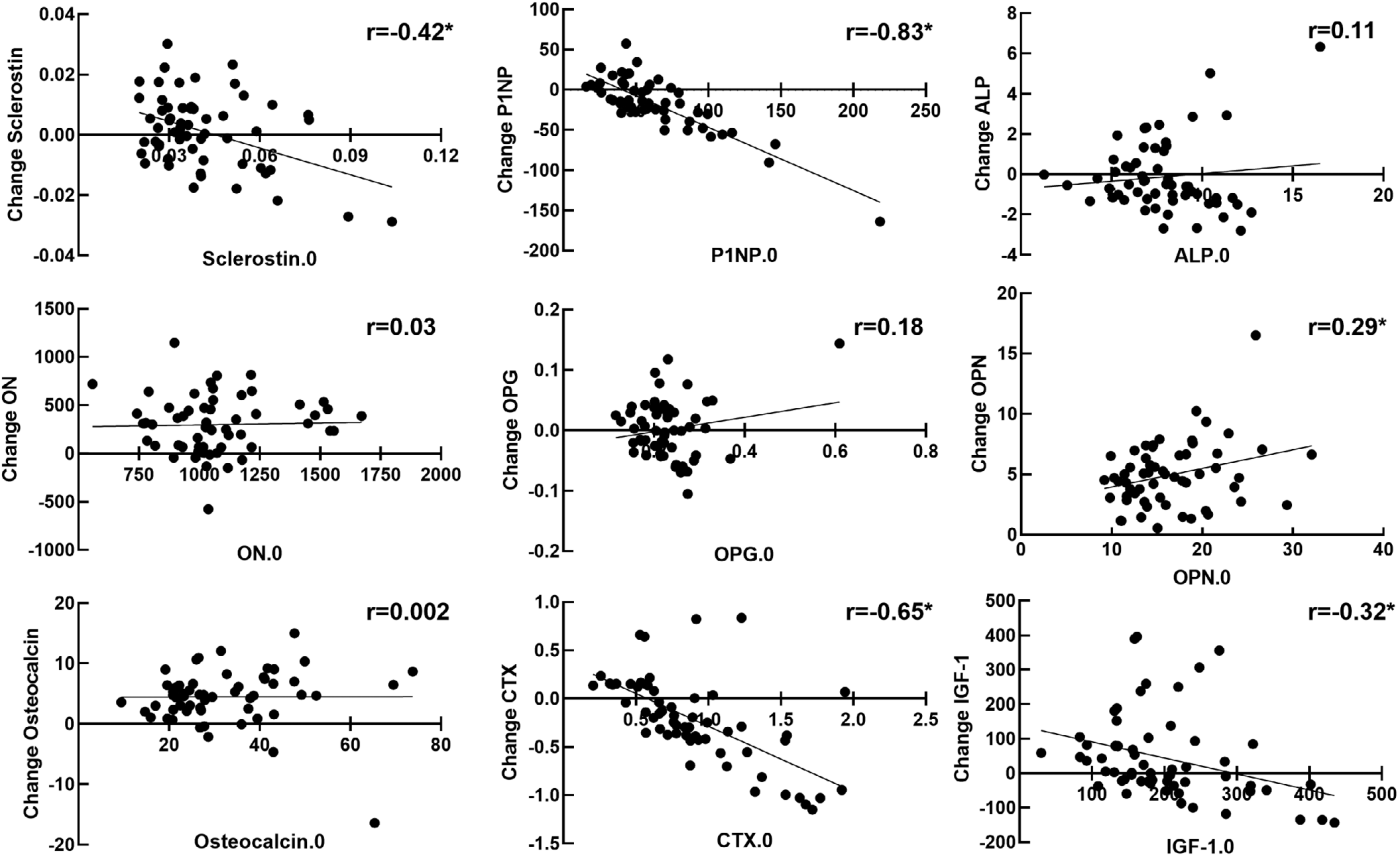
Correlation matrix of baseline BTMs versus the magnitude of change in BTMs during the clamp. *n* = 58 *p < .05 significant correlation.

Baseline ON (*r* = −0.30, *p* = .022) and OCN (*r* = −0.41, *p* = .002) were negatively correlated with age at T1D diagnosis, but baseline BTMs were not associated with HbA1c. During the HEC, insulin increased from 11.6 ± 8.3 to 50.6 ± 20.3 μU/mL. During the HEC, P1NP significantly decreased (−14.5 ± 44.3%, *p* = .020) from baseline. OCN, OPN, ON, and IGF‐1 all significantly increased (16.0 ± 13.1%, 31.3 ± 15.4%, 29.7 ± 31.7%, and 34.1 ± 71.2%, respectively; all *p* < .001) during the clamp (Fig. 1). The increase in sclerostin was not significant (7.3 ± 32.9%; range, −46% to +102%; *p* = .098), but there was a trend for a decrease in CTX (−12.4 ± 48.9; range, −67% to +125%; *p* = .058). ALP and OPG were not changed from baseline (*p* = .23 and *p* = .77). The magnitude of change in several BTMs (ALP, SCL, CTX, and P1NP) in response to hyperinsulinemia was negatively correlated with their baseline BTM values (Fig. 2): baseline versus change in ALP (*r* = −0.95); baseline versus change in SCL (*r* = −0.43); baseline versus change in CTX (*r* = −0.59); and baseline versus change in P1NP (*r* = −0.85). The positive correlation of baseline versus change in OPN was also significant (*r* = 0.29). The correlation between baseline IGF‐1 and the change in IGF‐1 neared significance IGF‐1 (*p* = .052, *r* = −0.33).

**Fig 3:**
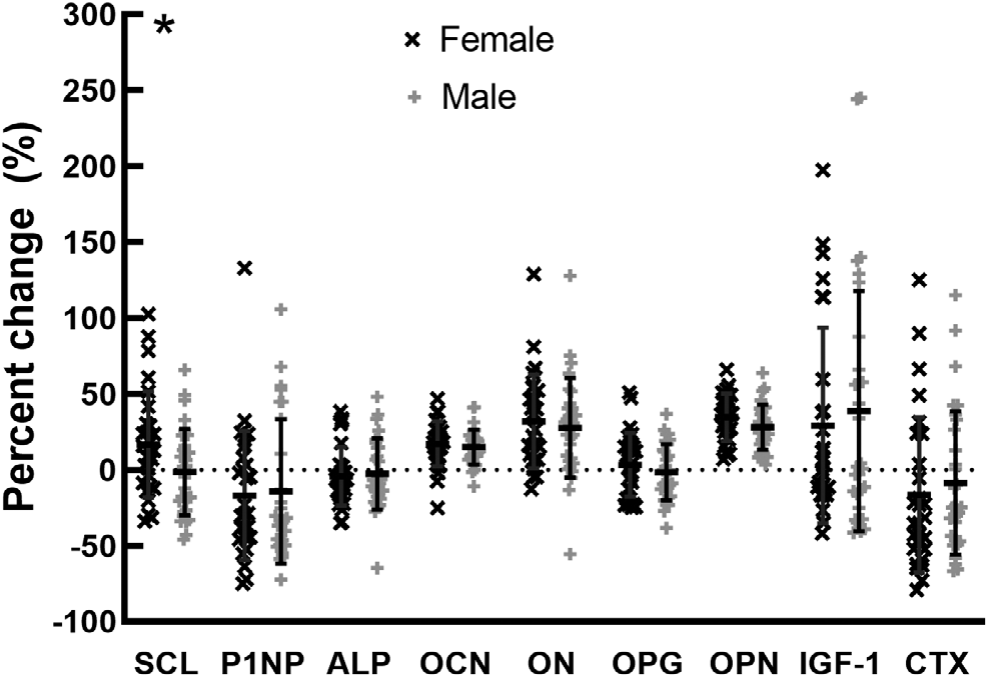
Sex comparisons of the relative (%) change in bone turnover markers in response to a hyperinsulinemic euglycemic clamp. Individual data points, with mean ± SD. Female *n* = 28; male *n* = 30. *p < .05 sex difference in response.

Neither the baseline BTMs nor the change in BTMs differed between C‐peptide groups (Tables 3 and 4). We did not detect significant correlations between changes in BTMs and HbA1c, or age at T1D diagnosis. Baseline ON and SCL were higher in men, but OPG was higher in women (Table 5). However, SCL was the only BTM that changed differently in women than men. The change in all other BTMs did not differ between sexes (Fig. 3).

**Table 3:** Baseline BTMs by C‐Peptide Status

BTM	Negative (*n* = 15)	Low (*n* = 14)	Medium (*n* = 12)	High (*n* = 17)	*p*
Sclerostin (ng/mL), mean ± SD	0.04 ± 0.02	0.04 ± 0.01	0.04 ± 0.02	0.05 ± 0.02	.57
P1NP (ng/mL), mean ± SD	66.47 ± 41.97	60.93 ± 22.51	62.43 ± 25.51	53.01 ± 24.27	.64
ALP (ng/mL), mean ± SD	6.83 ± 2.92	12.66 ± 16.17	7.39 ± 2.39	8.69 ± 2.94	.25
OCN (ng/mL), mean ± SD	32.49 ± 11.04	30.84 ± 9.72	33.06 ± 15069	31.46 ± 17.26	.98
ON (ng/mL; median [IQR])	1047.4 [915.4, 1167.4]	1059.5 [997.4, 1176.4]	1010.7 [925.0, 1054.6]	1019.7 [979.9, 1151.7]	.79
OPG (ng/mL), mean ± SD	0.21 ± 0.05	0.22 ± 0.06	0.23 ± 0.05	0.25 ± 0.10	.44
IGF (ng/mL; median [IQR])	181.0 [146.2, 226.5]	178.9 [132.8, 217.6]	188.7 [138.6, 250.4]	177.8 [154.8, 228.6]	.98
CTX (ng/L), mean ± SD	0.77 ± 0.37	0.85 ± 0.39	1.06 ± 0.53	0.97 ± 0.43	.29

**Table 4:** Relative Changes in BTMs by C‐Peptide Status

BTM	Negative (*n* = 15)	Low (*n* = 14)	Intermediate (*n* = 12)	High (*n* = 17)	*p*
Sclerostin ∆%	21.31 ± 29.29	−0.69 ± 35.97	−3.60 ± 20.11	9.04 ± 37.84	.177
P1NP ∆%	−19.25 ± 33.44	1.59 ± 59.18	−16.89 ± 54.19	−21.51 ± 32.04	.518
ALP ∆%	−10.67 ± 9.43	−4.82 ± 24.30	1.09 ± 21.55	0.84 ± 25.87	.402
OCN ∆%	14.28 ± 6.83	13.16 ± 14.42	18.97 ± 10.48	17.66 ± 17.51	.625
ON ∆%	35.06 ± 47.74	33.18 ± 26.47	21.91 ± 19.36	27.47 ± 25.90	.715
OPG ∆%	0.75 ± 13.15	−0.77 ± 20.63	1.69 ± 23.92	1.34 ± 22.23	.989
IGF ∆%	3.50 ± 61.92	27.06 ± 60.14	62.84 ± 91.05	46.61 ± 68.78	.151
CTX ∆%	−9.03 ± 36.25	−13.79 ± 65.17	−11.99 ± 46.12	−14.62 ± 49.33	.990

Values are mean ± SD.

**Table 5:** Baseline BTMs by Sex

BTM	Female (*n* = 28)	Male (*n* = 30)	*p*
Sclerostin (ng/mL), mean ± SD	0.03 ± 0.01	0.05 ± 0.02	<.001
P1NP (ng/mL), mean ± SD	60.26 ± 27.93	60.23 ± 30.67	.997
ALP (ng/mL), mean ± SD	7.63 ± 2.96	10.08 ± 11.27	.271
OCN (ng/mL), mean ± SD	28.82 ± 12.01	34.79 ± 14.18	.090
ON (ng/mL), median [IQR]	995.3 [814.3, 1060.9]	1061.7 [1007.9, 1206.8]	.010
OPG (ng/mL), mean ± SD	0.25 ± 0.09	0.21 ± 0.05	.029
IGF (ng/mL), median [IQR]	196.8 [164.0, 250.3]	160.4 [137.0, 219.7]	.219
CTX (ng/L), mean ± SD	0.83 ± 0.33	0.98 ± 0.50	.189

## Discussion

We examined acute bone turnover response to an HEC in adults with T1D with and without stratification by residual C‐peptide status and sex. Exogenous insulin acutely influenced markers of bone turnover activity during euglycemia in adults with T1D in a manner that suggests that, in most, bone resorption was mildly suppressed, and the effect on bone formation depended on the marker of interest. Contrary to our hypothesis, the acute bone turnover response to hyperinsulinemia did not depend on β‐cell function, based on the C‐peptide response to a MMTT. Although there were sex differences in resting BTM concentrations, SCL was the only BTM that demonstrated a sex difference in the response to an HEC.

All prior investigations into the effect of T1D on BTMs have relied on cross‐sectional comparisons of resting concentrations against either healthy controls or those with type 2 diabetes, with a paucity of cohort studies.^(^ ^27^, ^28^, ^29^, ^30^ ^)^ Additionally, most comparisons of BTMs have been in children and adolescents, with the aim of associating hyperglycemia with impaired bone accrual and increased risk of fracture in T1D.^(^ ^27^ ^)^ We measured several markers of bone turnover to gain a more comprehensive understanding of the acute effect of exogenous insulin on bone remodeling. An increase in SCL would be expected to precede the decrease in P1NP, and although the increase in SCL during the clamp was not significant, previous cross‐sectional studies associated increased SCL with having T1D.^(^ ^21^, ^22^, ^31^ ^)^ The decrease in P1NP indicates that hyperinsulinemia can acutely suppress type I collagen deposition in bone. In contrast, an increase in ON would indicate increased binding with calcium and an increase in mineralization. To our knowledge, we are the first to report ON responses in those with T1D. A repeated, combined decrease in collagen formation and an increase in mineralization could reduce the toughness of bone, increasing the susceptibility to fracture from a fall. Such a mechanism would be consistent with increased mineralization in those with T2D and in those with fracture and T1D, but mechanistic studies would be needed to fully interrogate this link.^(^ ^32^, ^33^ ^)^ Studies have reported no differences in spine BMD and modestly reduced BMD measured by DXA between subjects with T1D and matched controls despite higher fracture risk in T1D, suggesting that normal to increased mineralization results in compromised bone quality.^(^ ^34^, ^35^ ^)^ These results also suggest that hyperinsulinemia may influence osteoblast function both directly and indirectly via insulin action in osteocytes.

Because of the feedforward relationship between pancreas and bone, we expected to see a difference in the BTM response between C‐peptide groups. Our results suggest that residual β‐cell function does not alter the skeletal responsiveness to exogenously administered insulin. The increase in OCN was in line with preclinical evidence that insulin promotes the secretion of OCN.^(^ ^36^, ^37^ ^)^ Osteoclasts are responsible for decarboxylation, converting OCN into its more metabolically active form, those levels will be dependent on total OCN. Although the decrease in CTX was not significant, a decrease in osteoclast activity would then decrease the amount of decarboxylated OCN that reaches circulation. These results do contrast with the responses in those with type 2 diabetes and obese, insulin‐resistant individuals, in which P1NP, CTX and OCN were not altered during an HEC clamp.^(^ ^38^ ^)^ Further, both OCN and CTX were decreased in those who were insulin sensitive obese and lean. Two subjects with type 2 diabetes were treated with only lifestyle modification and five subjects received sulfonylurea and metformin. Hyperinsulinemia associated with obesity, type 2 diabetes, and sulfonylurea treatment may have blunted further effect of insulin treatment in these subjects.^(^ ^38^ ^)^ There is accumulating evidence that the suppression of bone turnover in response to a glucose challenge is less robust with increased insulin resistance and the presence of type 2 diabetes.^(^ ^8^, ^39^, ^40^ ^)^

The underlying mechanism for decreased osteoclast activity may be related to elevated OPG activity, because OPG inhibits osteoclastogenesis by acting as a decoy receptor for RANKL. Elevated circulating OPG has been reported in those with T1D as compared to healthy controls, but the contributors to OPG in circulation are not limited to bone.^(^ ^41^, ^42^, ^43^ ^)^ Importantly, OPG has also been associated with cardiovascular risk, ulcer development, and peripheral neuropathies.^(^ ^44^, ^45^, ^46^, ^47^ ^)^ The mechanism by which OPG contributes to these conditions may be through the inhibition of RANKL‐induced angiogenesis, although OPG may also promote angiogenesis.^(^ ^48^, ^49^ ^)^ Because OPG did not change in response to the HEC, our results indicate that exogenous insulin does not acutely contribute to potential OPG‐induced alterations in neurovascular function or bone turnover, but the source of increased OPG in T1D would need to be isolated to better inform its potential role in T1D‐related complications. It is also important to note that subjects in our study were young premenopausal females and the effect of insulin on OPG levels in young females may be different compared to postmenopausal women.

An important strength of this study was a larger sample size for a T1D trial, inclusion of patients with various beta cell function, and the inclusion of sex comparisons on the BTM to insulin. Many studies of BTMs in those with T1D have been limited to one sex, or with the sexes combined within the analysis of the primary outcome. Considering the known sex differences in fracture risk across the lifespan, we chose to examine sex differences in the BTM response to insulin. We were underpowered to perform sex comparisons within each C‐peptide group (ie, a 2 × 4 design), but considering the lack of sex differences across C‐peptide groups and the lack of differences between the C‐peptide groups, it is unlikely that these differences are large or even present with premenopausal women and men. Although the responses to insulin were similar between sexes for most bone markers when comparing premenopausal women against men, it is possible that sex differences could emerge in postmenopausal women. Bone turnover rates become more rapid and more uncoupled during the menopausal transition, and it is not yet clear how T1D interacts with the changes in metabolic function that occur with the loss of estrogen. However, we did observe the negative association between baseline BTMs and the change in BTMs, so it is possible that BTMs would change less in postmenopausal women who have a higher bone turnover rate.

This study represents an important step in uncovering a mechanistic basis by which T1D negatively affects bone quality and increases fracture risk across the lifespan in humans. HbA1c is a common correlate against fasting markers of bone turnover, particularly with diabetes, but HbA1c was not correlated with the BTM response to acute hyperinsulinemia. This suggests that chronic glycemic control was not correlated with acute BTM responses to insulin. Similarly, the age at T1D diagnosis was not associated with the acute BTM response, but the age at diagnosis is an independent determinant of bone quality in adulthood.^(^ ^2^ ^)^ If the chronic metabolic state does not influence acute BTM responses to metabolic perturbation, an important question remains about how acute bone turnover responses to a metabolic perturbation translate to bone quality outcomes. The HEC is used to assess insulin sensitivity, whereby models and assumptions are focused on the skeletal muscle, liver, and adipose tissue response to insulin. During the clamp we infuse glucose to maintain blood glucose levels, but little is known about the kinetics of glucose disposal in bone in humans during a clamp, and whether kinetics in bone are similar to muscle. Because we measured at baseline and at 120 min, we were not able to test how quickly or for how long BTMs remain altered in response to 2 hours of hyperinsulinemia. Responses to insulin reaching postprandial levels via intravenous route might be more robust than would be expected from a subcutaneous administration, which is a more common route of insulin delivery in treating T1D due to differences in kinetics. Either way, it is unclear whether BTMs that have been acutely altered by insulin would have a similar or potentiated response to a subsequent dose of insulin. Therefore, clinical implication of findings of study in patients with T1D on chronic long‐acting and short‐acting subcutaneous insulin therapy needs further investigation.

## Conclusion

Acute hyperinsulinemia has an immediate effect on bone turnover markers in T1D, but the response does not depend on pancreatic β‐cell function and was not related to glycemic control. The bone turnover response to exogenous insulin also does not appear to be sex dependent. Future work is needed to determine whether repeated doses of insulin induce a prolonged suppression of bone turnover and increase fracture risk.

## Disclosures

VNS reports receiving honoraria from Sanofi US, Medscape, and Dexcom Inc through the University of Colorado Denver and grants from vTv Therapeutics, Mylan GmbH, NovoNordisk, Sanofi US, and Juvenile Diabetes Research Foundation, outside the submitted work. MRR reports receiving honoraria from Semma Therapeutics and grant support from Xeris Pharmaceuticals, outside the submitted work. VDS, TV, LP, JKS, KJN, KMM, and CJN have nothing to disclose.

## AUTHOR CONTRIBUTIONS

**Vanessa Sherk:** Writing‐original draft; formal analysis; writing‐review and editing. **Timothy Vigers:** Data curation; formal analysis; writing‐review and editing. **Laura Pyle:** Data curation; formal analysis; writing‐review and editing. **Janet Snell‐Bergeon:** Data curation; formal analysis; methodology; writing‐review and editing. **Kristen Nadeau:** Conceptualization; investigation; methodology; writing‐review and editing. **Michael Rickels:** Conceptualization; data curation; investigation; methodology; project administration; writing‐review and editing. **Kellee Miller:** Data curation; methodology; project administration; resources; supervision; writing‐review and editing. **Carla Greenbaum:** Conceptualization; investigation; methodology; project administration; writing‐review and editing.

### Peer Review

The peer review history for this article is available at https://publons.com/publon/10.1002/jbm4.10389.

## Supporting information


**Supplemental Table 1.** Full cohort characteristics

**Supplementary Appendix**. T1D Exchange β‐Cell Function Study Group principal investigators (PI), co‐investigators (I), statisticians (S), and coordinators (C)
