# Supplementary material for: Acute Hyperinsulinemia Alters Bone Turnover in Women and Men With Type 1 Diabetes
Source: JBMR Plus. 2020 Aug 3;4(9):e10389. doi: 10.1002/jbm4.10389 (PMC7507374; doi:10.1002/jbm4.10389)
Supplement: Supplementary file 1 — Supplemental Table 1. Full cohort characteristics [file JBM4-4-e10389-s001.docx]

**Supplemental Table 1**. Full cohort characteristics

|  | Overall (n=58) |
| --- | --- |
| Age (y) | 27.8 (9.3) |
| Gender = M (%) | 30 (51.7) |
| Ethnicity (%) |  |
| Not Hispanic or Latino | 54 (93.1) |
| Hispanic or Latino | 3 ( 5.2) |
| Unknown/not reported | 1 ( 1.7) |
| Race (%) |  |
| White | 54 (93.1) |
| Asian | 1 ( 1.7) |
| Black/African American | 1 ( 1.7) |
| More than one race | 1 ( 1.7) |
| Unknown/not reported | 1 ( 1.7) |
| T1D Duration (median [IQR]) | 7.4 (6.5) |
| BMI (kg/m^2^) | 24.2 (3.1) |
| Daily insulin (Units/kg) | 0.58 (0.22) |
| HbA1c (%)^a^ | 7.0 (0.9) |
| Insulin Pump Use (%) | 34 (58.6) |
| CGM Use = Yes (%) | 25 (43.1) |
| Vitamin D (IU) | 26.5 (7.5) |
| Calcium (mg/dL) | 9.34 (0.35) |
| Albumin (g/dL) | 4.40 (0.25) |
| Ca_Adjusted ^b^ | 9.42 (0.34) |
| eGFR (mL/min/1.73m^2^) ^c^ | 108.9 (22.7) |
| Creatinine (mg/dL) | 0.82 (0.15) |

Mean (SD) unless otherwise stated.

^a^To convert to mmol/mol, multiply by 10.93 and subtract 23.50.

^b^ Corrected Calcium = (0.8 * (Normal Albumin - Pt's Albumin)) + Serum Ca. Normal albumin was defaulted to 4 mg/dL.

^c^ eGFR was calculated using MDRD equation.
