# Supplementary material for: Acute Hyperinsulinemia Alters Bone Turnover in Women and Men With Type 1 Diabetes
Source: JBMR Plus. 2020 Aug 3;4(9):e10389. doi: 10.1002/jbm4.10389 (PMC7507374; doi:10.1002/jbm4.10389)
Supplement: Supplementary file 2 — Supplementary Appendix. T1D Exchange β‐Cell Function Study Group principal investigators (PI), co‐investigators (I), statisticians (S), and coordinators (C) [file JBM4-4-e10389-s002.docx]

**Supplementary Material**

**T1D Exchange β-Cell Function Study Group principal investigators (PI), co-investigators (I), statisticians (S), and coordinators (C):**

Barbara Davis Center for Childhood Diabetes and Children’s Hospital Colorado, University of Colorado School of Medicine, Aurora, CO (n =14): Viral N. Shah (Co-PI), Kristen J. Nadeau (Co-PI), Melanie C. Green (I); Jayne Williams (I), Amy Baumgartner (C);

Benaroya Research Institute, Seattle, WA (n =12): Carla J. Greenbaum (PI), Wei Hao (I), Henry T. Bahnson (S), Alyssa Ylescupidez (S);

Children’s Mercy Hospital, Kansas City, MO (n =11): Mark A. Clements (PI);

Institute for Diabetes, Obesity & Metabolism, University of Pennsylvania Perelman School of Medicine, Philadelphia, PA (n =10): Michael R. Rickels (PI), Amy J. Peleckis (I), Cornelia Dalton-Bakes (C);

Yale University School of Medicine, New Haven, CN (n =9): Jennifer Sherr (PI); Eileen Tichy (I), Kate Weyman (I), Melinda Zgorski (C), Amy Steffen (C);

AdventHealth Translational Research Institute for Metabolism and Diabetes, Orlando, FL (n =5): Richard E. Pratley (PI);

Center for Diabetes and Metabolic Disease, Indiana University School of Medicine, Indianapolis, IN (n =2): Tamara S. Hannon (PI), Carmella Evans-Molina (I);

Jaeb Center for Health Research, Tampa, FL: Kellee M. Miller (S).
